# Impact of Symptomatic Slow-Acting Drugs on Inflammatory Pathways in Osteoarthritis: Therapeutic Advances and Future Challenges

**DOI:** 10.1021/acsptsci.5c00521

**Published:** 2025-11-18

**Authors:** Vitor Alfredo de Santana Silva, Katarine Gabriely Aurista do Nascimento, Priscila Gubert, Maria G. Carneiro-da-Cunha, Kátia Alves Ribeiro, Paulo Antônio Galindo Soares

**Affiliations:** † Department of Biochemistry/Keizo Asami Institute-iLIKA, Federal University of Pernambuco (UFPE), Av. Prof. Moraes Rego, s/n, Cidade Universitária, CEP: 50670-420 Recife, Pernambuco, Brazil; ‡ Postgraduate Program in Biology Applied to Health, PPGBAS, 28116 Federal University of Pernambuco, CEP: 50670-901 Recife, Pernambuco, Brazil

**Keywords:** chondroitin sulfate, glucosamine, hyaluronic acid, inflammation

## Abstract

Osteoarthritis (OA) is a leading cause of physical disability, psychological distress, and a significant economic burden worldwide. Current treatments alleviate symptoms; however, disease progression remains largely uncontrolled, highlighting the urgent need for investigation of disease-modifying therapies. Symptomatic slow-acting drugs for osteoarthritis (SYSADOAs), such as glucosamine (GlcN), chondroitin sulfate (CS), and hyaluronic acid (HA), have gained increasing attention for their potential benefits in alleviating pain and mitigating the inflammatory and degenerative processes that characterize OA. These compounds modulate several homeostatic mechanisms, promoting anti-inflammatory, antioxidant, antiapoptotic, and anabolic countermensuring effects. Nevertheless, debates regarding their long-term efficacy and safety remain controversial, which explains why major osteoarthritis societies do not provide the same recommendations for the pharmacological treatment of OA. In this context, this review critically evaluates the current evidence surrounding HA, GlcN, and CS, highlighting their safety, mechanisms of action, and promising therapeutic perspectives for modifying the natural course of knee, hand, and hip OA.

## Graphical abstract

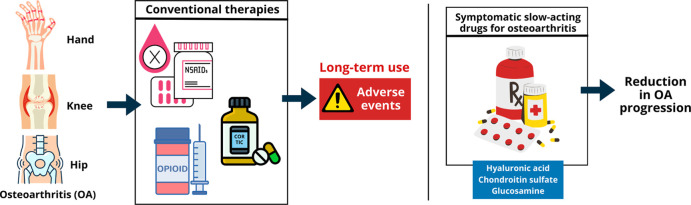

Osteoarthritis (OA) is a highly prevalent musculoskeletal rheumatic disorder that significantly impacts individual health, healthcare systems, and overall socioeconomic costs.^1^ OA remains a substantial global health burden: in 2020, an estimated 595 million people (≈7.6% of the global population) had OA, representing a 132.2% increase in cases since 1990. Prevalence rises steeply with age. Age-standardized prevalence in 2020 was higher in women (8058.9 per 100,000) than in men (5780.1 per 100,000). The knee was the most commonly affected joint, with an age-standardized prevalence of 4307.4 per 100,000 in 2020.^2^

Although aging is a major risk factor, OA is not an inevitable consequence of growing older; modifiable factors include obesity (high body mass index), prior joint injuries, sarcopenia, and occupations involving repetitive strain.^2,3^ By 2050, global OA cases are projected to increase by 60 to 100%, driven by aging populations and rising obesity rates.^2^ Notably, high BMI alone has been attributed to 20% of the rise in OA-related disability, and OA currently ranks as the 11th leading cause of global disability.^2,3^

The most commonly used drug therapies for pain relief and improvement of joint function are nonsteroidal anti-inflammatory drugs (NSAIDs), intra-articular corticosteroids and opioids, and slow-acting symptomatic medications for OA (SYSADOA); although NSAIDs are the most prescribed according to international recommendations, especially in elderly patients due to their efficacy and lack of dependence, their prolonged use significantly increases the risk of serious adverse events (e.g., gastrointestinal, cardiovascular, and renal toxicity).^4^

In response to the therapeutic need for OA treatment, SYSADOAs have been proposed as chondroprotective agents capable of slowing, stabilizing, or even reversing pathological changes in osteoarthritic joints, thereby limiting disease progression.^5^ Among the most studied SYSADOAs are hyaluronic acid, chondroitin sulfate, glucosamine, and diacerein.^6^ Each agent presents unique characteristics, with some offering symptomatic relief while others demonstrating potential disease-modifying benefits. Hyaluronic acid, chondroitin sulfate, and glucosamine are the most commonly used and discussed compounds in the literature, having been the subject of various clinical and experimental studies.^5,7^

Hyaluronic acid (HA) is a linear anionic polysaccharide of the glycosaminoglycan family, composed of distinct repetitive units of β-1,4-d-glucuronic acid (uronic acid) and β-1,3-d-*N*-acetylglucosamine. It is the only naturally occurring nonsulfated glycosaminoglycan (Figure 1A). The Osteoarthritis Research Society International (OARSI) has conditionally recommended the use of intra-articular hyaluronic acid (IAHA) for the treatment of knee OA, regardless of long-term comorbidity groups.^8^

**Figure 1:**
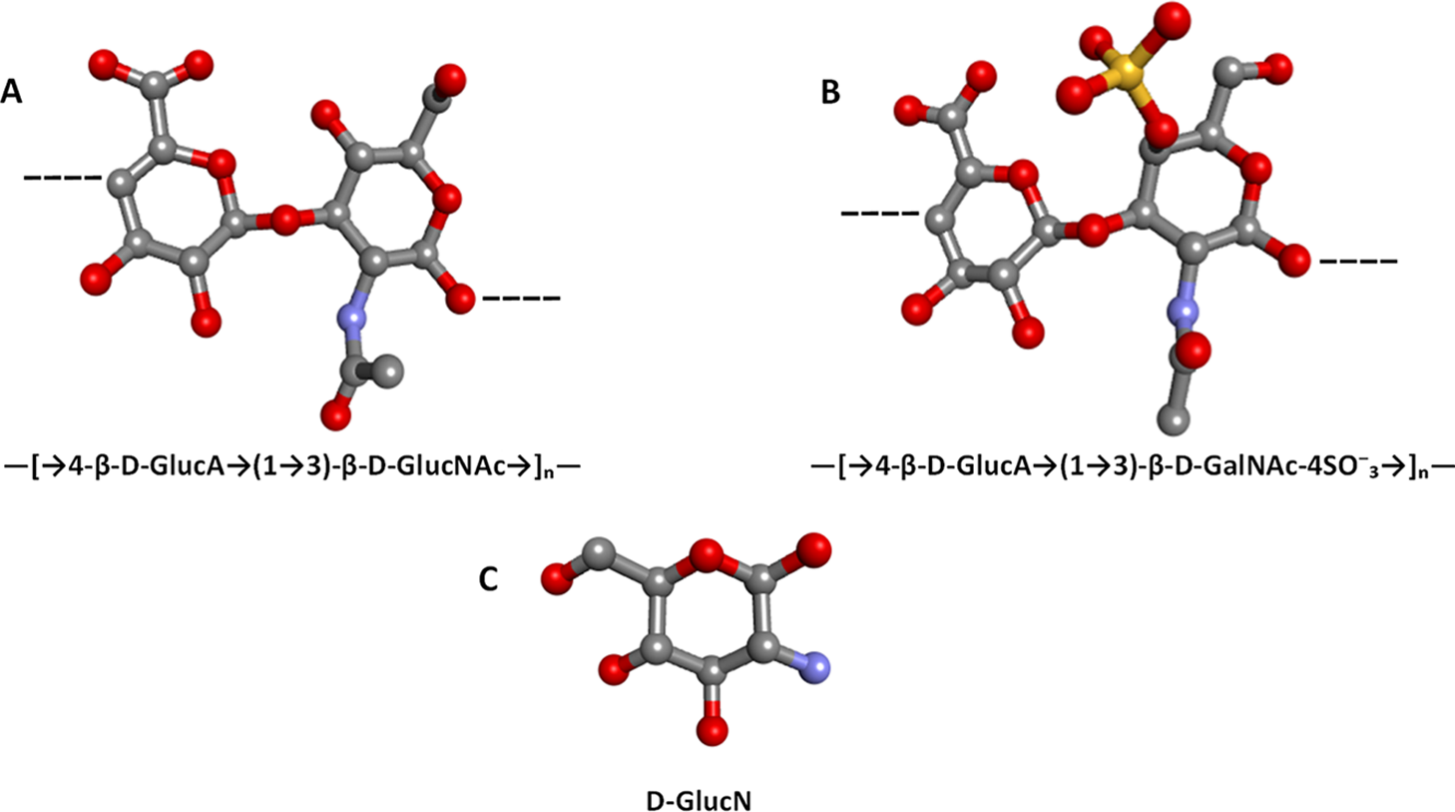
Three-dimensional stick models of glycosaminoglycan (GAG) disaccharide repeats and a glucosamine monomer, drawn in PubChem and exported as SMILES, then rendered in Discovery Studio. (A) Hyaluronic acid (―[→4-β-d-GlucA → (1 → 3)-β-d-GlcNAc→]_*n*_―); (B) chondroitin-4-sulfate (CS-A) (―[→4-β-d-GlucA → (1 → 3)-β-d-GalNAc-4-SO_3_^–^→]_*n*_―); (C) glucosamine monomer (d-GlcN). In each stick model, carbon is colored gray, nitrogen blue, hydrogen light gray, oxygen red, and sulfur yellow. Hydrogen atoms have been omitted for clarity.

Chondroitin sulfate (CS) is an anionic, sulfated glycosaminoglycan found in various connective tissues, including cartilage, bone, ligaments, tendons, and skin. CS has been shown to exert beneficial effects in OA by modulating inflammatory and catabolic factors while promoting the production of anabolic factors. It is a linear heteropolysaccharide with gel-like properties, such as lubrication, water retention, and load resistance, and accounts for approximately 80% of all glycosaminoglycans in articular cartilage. The molecular weights of naturally occurring chondroitin sulfates range from 10 to 100 kDas (kDa).^9,10^ Structurally, it is a sulfated polysaccharide composed of repetitive units of β-1,3-d-glucuronic acid and β-1,4-d-*N*-acetylgalactosamine (GalNAc) and is broadly classified based on the sulfation pattern (Figure 1B). In terrestrial animals, it is predominantly found as monosulfated disaccharides at the 4-O or 6-O positions of GalNAc (CS-A and CS-C, respectively), along with a small percentage of nonsulfated disaccharides (CS-0). Studies have shown that the specific distribution of sulfate throughout its structure can impact its biological activities.^11,12^

Glucosamine (2-amino-2-desoxi-d-glicose) (GlcN) is an amino-monosaccharide and a natural constituent of some GAGs (Figure 1C), serving as a substrate for the formation of molecules such as proteoglycans, HA, and CS in the articular cartilage.^13,14^

Compared to NSAIDs, the SYSADOAs discussed herein (GlcN, CS, and HA) generally demonstrate a more favorable safety profile while providing symptomatic relief, with certain formulations also showing potential for structural modification in OA.^15–17^ Supplementation with these biomolecules has been demonstrated to mediate various homeostatic processes in OA, promoting anti-inflammatory, antioxidant, antiapoptotic, anticatabolic, and anabolic effects.^16,17^ However, controversies regarding the efficacy and safety of these SYSADOAs persist, which is why major research institutions do not provide strong recommendations for the pharmacological treatment of OA. Although numerous studies have investigated therapies with clinically relevant effects, it remains necessary to explore specific molecular and phenotypic targets to develop more targeted and precise therapies, including HA, CS, GlcN, diacerein, herbal formulations such as SKCPT, and other classes of SYSADOAs, evaluated according to clinical phenotypes and biomarkers. This review provides a comprehensive and updated assessment of the safety and efficacy of the main SYSADOAs (HA, CS, GlcN) based on clinical, experimental, and meta-analytical studies involving particularly knee OA. While foundational and pioneering studies are acknowledged, particular emphasis is placed on the most recent evidence to capture the current state of knowledge, clarify ongoing controversies, and highlight the latest therapeutic advances. We conclude by discussing emerging approaches and future perspectives for OA treatment.

## Hyaluronic Acid in Osteoarthritis: Applications and Mechanisms

### Clinical Efficacy and Safety Profile of Intra-articular HA

Hyaluronic acid (HA) is present in all connective tissues and organs, and it is more abundant in joint cartilage and synovial fluid. Its role is to contribute to cell proliferation, migration, and morphogenesis, lubrication and viscoelasticity of synovial fluid, as well as to act as a moisture retention, joint stabilizer, and water balance regulator.^18,19^ The therapeutic efficacy of HA preparations may differ depending on the HA source, molecular weight, production methods, dosage, rheological properties, concentration, joint half-life, pharmacokinetics, and pharmacodynamics.^20^ The 2024 meta-analysis published in Pharmaceuticals of 18 randomized controlled trials (*n* = 3851) demonstrated that intra-articular HA injection in the knee, compared to placebo, significantly reduced Western Ontario and McMaster Universities Osteoarthritis Index pain by 1.24 points (95% CI −1.78 to −0.70) and Western Ontario and McMaster Universities stiffness by 0.76 points (95% CI −1.34 to −0.18) at 2–4 weeks. No significant differences were observed for the analog visual scale at rest or the Western Ontario and McMaster Universities function. At 5–8 weeks, HA reduced the visual analog Scale at rest by 1.02 points (95% CI −1.79 to −0.24), with fewer than 4% reporting transient injection-site discomfort.^7^

In a 2024 double-blind randomized clinical trial (RCT) of 30 patients with knee OA, three weekly intra-articular knee injections of Hylan G-F 20 (a high-molecular-weight form of hyaluronic acid) reduced pain on a Analog Visual scale by 45.7 mm (from 62.6 ± 16.1 to 16.9 ± 9.7; *p* < 0.001) at 12 weeks and improved stiffness at 24 weeks (*p* < 0.01), with only 6.7% reporting mild local pain and no serious adverse events.^21^

A 2025 Bayesian network meta-analysis of 37 RCTs (*n* = 5089) confirmed medium-term pain relief, showing an average reduction of 0.30 standard deviations (95% credible interval −0.45 to −0.15) at six months and 0.25 standard deviations (95% credible interval −0.40 to −0.10) at 12 months; functional improvement showed standardized mean differences (SMD) of −0.18 (95% credible interval −0.32 to −0.04) at 6 months, attenuating to −0.10 (95% CrI −0.24 to 0.04) at 12 months.^22^ In the systematic review published in the British Medical Journal including 24 RCTs with 8997 participants, intra-articular HA injection for knee OA demonstrated modest but significant efficacy for pain at 6 months (SMD = −0.08; 95% CI −0.15 to −0.02), equivalent to −2.0 mm on visual analog scale, while the safety profile revealed a small increased risk of serious adverse events related to intra-articular HA treatment (relative risk 1.49; 95% CI 1.12–1.98) compared with placebo. The most frequently reported serious adverse events included joint infections requiring medical intervention, cardiovascular complications, and other conditions resulting in hospitalization. Although these events occurred in patients receiving viscosupplementation (a procedure synonymous with intra-articular HA injection), not all were directly attributable to the injection procedure itself.^23^

In 51 patients with knee OA, a single injection of HANOX-M-XL provided symptomatic relief for a mean of 52 weeks, with a range of 13–155 weeks, and 85.5% reported sustained clinical improvement. Patients with early stage osteoarthritis, defined as Kellgren–Lawrence grades 1 and 2, experienced relief 14 weeks longer than those with advanced disease, 62.6 ± 36.4 weeks versus 48.9 ± 18.6 weeks (*p* = 0.03). A lighter radiographic grade, older age, and male gender were linked to a more durable response (*p* = 0.007, *p* = 0.04, and *p* = 0.02, respectively). Safety was excellent with only mild local reactions and no serious adverse events reported.^24^ Table 1 provides a comparative summary of several clinical studies evaluating the use of HA in OA, highlighting the different dosages, study types, objectives, and outcomes of these investigations.

**Table 1:** Comparative Summary of Clinical Studies on the Use of Hyaluronic Acid in Knee Osteoarthritis^a^

study type	dosage	objective	sample	clinical outcomes	nutraceutical presentation
single-blinded, randomized controlled trial	HMW-HA: one 3 mL injection containing 60 mg HA (cross-linked)	to compare pain and functional outcomes of a single cross-linked HMW-HA injection versus three LMW-HA injections in knee OA patients	90 patients	both regimens produced significant and equivalent improvements in WOMAC, Lequesne, and analog visual scale scores at 2 and 6 months; only WOMAC stiffness favored LMW-HA at 2 months^25^	solution HMW-HA: 60 mg; LMW-HA: 20 mg
phase 3, multicenter, randomized,double-blind, placebo-controlled, parallel-group study	single intra-articular HA injection of 64 mg (32 mg high *M*_W_ + 32 mg low *M*_W_/2 mL)	evaluate the efficacy and safety of high and low molecular weight HA formulation vs placebo in knee OA	692 patients	significant pain reduction, better function, quality of life, and lower rescue medication in the HA-HL group^26^	solution (32 mg high *M*_W_ + 32 mg low *M*_W_/2 mL)
prospective,double-blind, RCT	single injection (20 mg/3 mL)	compare the efficacy of single PRP vs cross-linked HA in early knee OA	56 patients	WOMAC total ↓ 4.36 ± 1.56 (23.9% improvement); pain ↓ 1.59 ± 0.40; stiffness ↓ 0.43 ± 0.18; function ↓ 2.34 ± 1.17 ^27^	Solution (60 mg HA; 20 mg/mL)
double-blind randomized controlled trial	cross-linked hyaluronate: 1 single IA dose	evaluate the efficacy and safety of cross-linked hyaluronate vs placebo in patients with knee OA	128 patients	significant improvement in pain and joint function in the cross-linked hyaluronate group compared to placebo^28^	solution (20 mg/3 mL)
RCT	three weekly IA injections of PN or HMW-HA at 1 week intervals	compare the efficacy and safety of PN vs HMW-HA for knee OA	60 patients	HMW-HA improved pain, function, and quality of life in knee OA patients, with mild adverse events^29^	polynucleotide: solution (20 mg/2 mL) and HMW-HA: solution (20 mg/2 mL)
RCT	doses of 20 mg, 32 mg, and 48 mg of HMW-HA	compare different doses of HMW-HA in knee OA patients	100 patients	over six months, pain, stiffness, function, and quality of life improved^30^	HMW hyaluronic acid solution (20 mg/2 mL, 32 mg/2 mL, and 48 mg/2 mL)
retrospective, single-center clinical study with prospectively collected data	Group I—15 mg/mL (30 mg per syringe); Group II—30 mg/mL (60 mg per syringe)	compare triple low-dose vs single high-dose HMW hyaluronic acid injections over 12 months in knee OA patients	128 patients	triple low-dose injections achieved greater and more sustained pain relief and functional improvement over 12 months than the single high-dose injection^31^	solution (prefilled 2 mL syringe, total 90 mg)
RCT	five weekly intra-articular injections of 20 mg/2 mL (total 100 mg)	evaluate efficacy and tolerability of 5 injections vs placebo; primary outcome: change in visual analog scale (50 ft) W0 → W25	200 patients	visual analog scale (W0 → W25): mean decrease of 30.85 mm with Hyalgan vs 23.62 mm with placebo; between-group difference 8.07 mm (*p* = 0.002). Pain and function scores on the Western Ontario and McMaster Universities Osteoarthritis Index also improved significantly. The treatment was safe and well-tolerated^32^	solution (20 mg/2 mL; 500–730 kDa sodium hyaluronate)
RCT	single 6 mL intra-articular injection of Hylan G-F 20	to assess the efficacy and safety of a single 6 mL injection vs placebo over 26 weeks	253 patients	pain reduction (Western Ontario and McMaster Universities Osteoarthritis Index) and improved walking pain, patient global assessment, clinical observer global assessment; no significant Western Ontario and McMaster Universities Osteoarthritis Index function change; safe with repeat injection^33^	solution (Hylan G-F 20), cross-linked HA, 6000 kDa, 6 mL single dose
PRISMA-compliant systematic review of overlapping meta-analyses	2–6 mL per injection, 1–5 weekly injections (varied across RCTs)	to evaluate the efficacy and safety of IA-HA vs placebo for knee OA and identify the highest-quality evidence	12 meta-anal	HA effective for pain relief, function improvement, and PGA; no increased risk of adverse events; cochrane review (Bellamy et al.^34^) ranked as highest quality evidence^35^	solution (HMW, LMW, cross-linked; multiple formulations and regimens)
systematic review and meta-analysis (safety)	20 mg/2 mL; 32 mg/2 mL; 48 mg/2 mL; 60 mg/3 mL; 90 mg/3 mL; typical volumes 2–3 mL; 1–5 injections	assess safety of IA-HA vs IA saline in patients with knee OA: AEs, local AEs, SAEs, and withdrawals	35 RCTs	no difference in overall AEs, SAEs, withdrawals; increased nonserious local reactions with IAHA (14.5% vs 11.7%; RR = 1.21, *p* = 0.003); reactions transient^36^	solution (typical volume: 2–3 mL; 1–5 injections; dose range: 20–90 mg)
observational prospective single-arm study	90 mg in 3 mL of HMW-HA (single injection)	evaluate the efficacy and safety of cross-linked HA in knee OA	50 patients	significant clinical score improvement at 3 and 6 months; benefit reduction at 12 months; minimal side effects^37^	DIART ONE solution (90 mg/3 mL)

a HMW-HA—high-molecular-weight hyaluronic acid; LMW-HA—low-molecular-weight hyaluronic acid; RCT—randomized controlled trial; IA—intra-articular; WOMAC—Western Ontario and McMaster Universities Osteoarthritis Index; VAS—visual analog scale; QoL—quality of life; PRP—platelet-rich plasma; PN—polynucleotide; AEs—adverse events.

### Pharmacokinetics and the Impact of Formulation

There are currently two production processes for obtaining polymers of HA in commercial quantities: the extraction of animal tissues, typically from cockerel crests, and bacterial expression systems in *Streptococcus* through microbial fermentation.^38^ Depending on the formulation, HA can be administered intra-articularly to affected joints (most commonly the knee) or given orally for symptomatic management of OA. Because OA affects a limited number of joints (shoulder, elbow, knee, hip, hands, and feet), local treatment has been reported as more effective in relation to oral administration since it avoids systemic exposures and potential side effects.^23,39^ Currently, different injectable formulations of HA are available: preparations with low molecular weight (500–730 kDa), intermediate (800–2000 kDa), and high (∼6000 kDa).^40^ In the United States, HA injections for OA are regulated by the Food and Drug Administration (FDA) as medical devices.

Recent research on the pharmacokinetics of medium molecular weight hyaluronic acid (300–600 kDa) administered intravenously has demonstrated its distribution and therapeutic effects in adjuvant-induced arthritis (AIA) models. Intravenous administration resulted in a predominant distribution in inflamed joints, with a blood half-life exceeding 8 h, promoting its persistence. The study conducted by Šimek et al.^41^ highlighted the notable distribution of HA in inflamed joints, particularly in the ankle joints, with exogenous HA concentrations remaining elevated in the inflamed areas over time. Furthermore, the research emphasized that due to the increased vascular permeability observed in inflamed tissues, intravenously administered HA not only targeted the joints but also reached other tissues with inflammatory processes, such as muscles and adjacent structures. These findings suggest that intravenous administration of HA may provide an effective therapeutic approach for treating joint inflammation, while also offering benefits in other areas of the body affected by inflammatory processes.

### Mechanisms of Action: Receptor-Mediated Modulation of Inflammation

HA is a linear glycosaminoglycan that interacts with the glycoprotein lubricin (PRG4), contributing to a lubrication network that decreases friction and reduces wear on joint surfaces.^42^ It was reported that altered characteristics of HA in the synovium, including decreased synthesis and increased degradation, as well as increased oxidative stress, lead to a decrease in the concentration and average molecular weight of HA, accompanied by the production of pro-inflammatory cytokines from chondrocytes, fibroblasts, and macrophages. The administration of HMW-HA in synovial joints seems to have the opposite effect on these molecular signaling pathways.^43^

HA can perform biological functions through two basic mechanisms: as a passive structural molecule and/or a signaling molecule (Figure 2). The passive mechanism is related to the physicochemical properties of HMW-HA (osmotic balance, viscoelasticity, and physical properties of ECM), while the signaling mechanism is related to pro- and anti-inflammatory activities (promotion and inhibition of cell migration, activation and blocking of cell division and differentiation). The signaling molecule function depends on the molecular weight of HA, location, and cell-specific factors (receptor expression, signaling pathways, and cell cycle). In addition, depending on the molecular weight of HA, there may be an influence on HA uptake by cells, changes in receptor affinity, and the formation of receptor complex clusters in targeted cells.^44^

**Figure 2:**
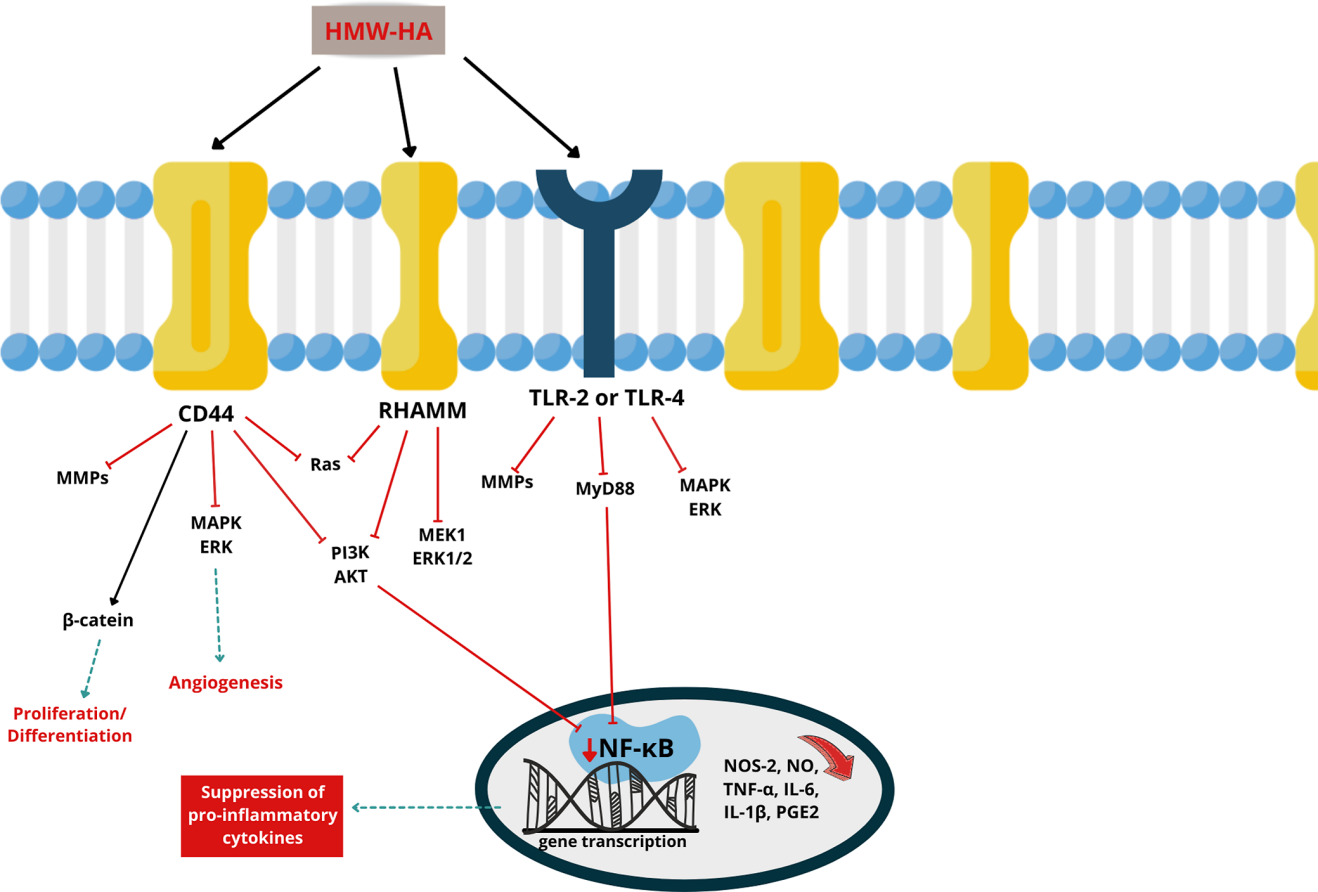
OA inflammatory pathways and anti-inflammatory activity of HMW-HA. HA: hyaluronic acid. HMW-HA: high molecular weight hyaluronic acid. CD44: cluster of differentiation 44. RHAMM: receptor for hyaluronic acid-mediated motility. TLR-2/TLR-4: toll-like receptor 2/toll-like receptor 4. MAPK: mitogen-activated protein kinase. ERK (ERK1/2): extracellular signal-regulated kinase (1/2). PI3K: phosphoinositide 3-kinase. AKT: protein kinase B (PKB). NF-κB: nuclear factor kappa-light-chain-enhancer of activated B cells. MMPs: matrix metalloproteinases. MyD88: myeloid differentiation primary response 88. β-Catenin: beta-catenin.

Table 2 presents the main receptors involved in hyaluronic acid’s biological activities, indicating their cellular localization and mechanism of action. The primary receptor for the HA ligand is the CD44 receptor, a type 1, multidomain, and multi-isoform transmembrane glycoprotein. CD44 is responsible for maintaining cartilage homeostasis, and its main function is to bind and internalize exogenous HA, which interacts with various ligands, including extracellular matrix proteins, osteopontin, collagens, matrix metalloproteinases (MMPs), growth factors, cytokines, and fibronectin.^45^

**Table 2:** Summary of Receptors Involved in Hyaluronic Acid Biological Activities^a^

receptors	location	mechanism
CD44	cell membrane (especially in lipid rafts), microvilli	controls internalization and degradation of AH, inflammation, angiogenesis, cell migration, proliferation, aggregation, and adhesion to ECM components^53^
TLR-2/4	cell membrane	involved in PAMP recognition^54^
layilin (LAYN)	cell membrane	regulates focal adhesions, controls cell–cell interactions, and modulates cell behaviors and function^46^
ICAM-1	cell membrane	induced during inflammatory responses^44^
RHAMM	cell membrane, cytoplasm, and nucleus	involved in cell motility, cell transformation, metastasis formation, regulation of adipogenesis, and extracellular regulated kinase (ERK) activity^55^
LYVE-1	cell membrane and vesicles near the extranuclear membranes	transports HA for degradation and aids in leukocyte adhesion and migration through the lymphatic endothelium^56^
HARE/Stabilin-2	cell membrane and cytoplasm	acts on the systemic clearance of HA and other GAGs, such as the phosphatidylserine receptor, which increases the involvement of apoptotic cells^57^

a HA—hyaluronic acid; CD44—cluster of differentiation 44; PAMP—pathogen-associated molecular pattern; ECM—extracellular matrix; TLR—toll-like receptor; LAYN—layilin; ICAM-1—intercellular adhesion molecule-1; RHAMM—receptor for hyaluronic acid-mediated motility; LYVE-1—lymphatic vessel endothelial hyaluronic acid receptor-1; HARE—hyaluronic acid receptor for endocytosis (also known as Stabilin-2).

The biological activity of HA is not exclusive to high-molecular-weight forms. Evidence suggests that low-molecular-weight HA (LMW-HA) and its oligosaccharides may also confer therapeutic benefits. For instance, clinical studies have shown that both a single injection of HMW-HA and a course of multiple LMW-HA injections can improve pain and function in OA patients, with some regimens offering specific advantages such as greater reduction in stiffness.^24^ Furthermore, in vitro models demonstrate that LMW-HA-derived oligosaccharides can mitigate inflammation, apoptosis, and extracellular matrix remodeling in chondrocyte cultures, highlighting their therapeutic potential without inducing cytotoxicity or pro-inflammatory effects in those experimental conditions.^46^ HMW-HA generally has anti-inflammatory effects and may even inhibit NF-κB. The current hypothesis explaining the effect of HWM-HA is that it forms groups of receptors on the cell membrane surface to modulate receptor activity. Thus, the binding of HA to the CD44 receptor provides a membrane-coating effect. This protective layer of HA on the cell surface can mask cell death receptors and prevent the cell from going into apoptosis.^44^

The activation of the HA-CD44 interaction is also involved in several intracellular signaling pathways that mediate cell invasion through the activation of nonphosphorylated β-catenin by the complex formed with GSK3 kinase and APC, thereby increasing its accumulation and promoting cell proliferation. HA-CD44 also stimulates cell proliferation and angiogenesis through the modulation of the MAPK/ERK signaling pathway and enhances PI3K/AKT activation, leading to the translocation of nuclear factor kappa B (NF-κB) and the production of inflammatory cytokines, among other processes such a\s HA internalization and degradation, aggregation, and adhesion to ECM components, Depending on the molecular weight of HA and the cellular context^45,47,48^

The interaction of HA with RHAMM, Toll-Like Receptor (TLR) 4/2, LAYN, ICAM-1, and HARE is also very important and triggers diverse signaling pathways. RHAMM-HA modulates the formation of extracellular signal-regulated kinase 2 (ERK2), MEK1, and ERK1/2 complexes, controls the activation and targeting of ERK1/2 to specific substrates, and the activation of Ras.^49^ TLR4/2-HA can promote the expression of matrix MMP in macrophages; it may activate chemokine genes, strongly dependent on the presence of MyD88, and in dendritic cells, it can induce cell maturation through the MAPK phosphorylation cascade and nuclear translocation of NF-κB, eventually leading to the production of TNF-alpha and other pro-inflammatory cytokines. The interaction between ICAM-1 and HA prevents the adhesion of other receptors, such as Mac-1.^50,51^

However, the interaction between LAYN-HA and LYVE-1-HA, as well as the intracellular pathways regulated by these receptors, remains partly unknown. Although their critical role is recognized, further investigations are still required regarding the signaling pathways involved.^50,52^

Toll-like receptors (TLRs) are a group of highly conserved proteins that coordinate initial defense against common pathogens within the immune system. The human TLR family consists of more than 10 receptors that can be activated by pathogen-associated molecular patterns (PAMPs) and damage-associated molecular patterns (DAMPs).^54^ Two mechanisms are proposed to explain how HA can influence TLRs: (1) in the first theory, LWM-HA acts as an agonist for TLR-2 and TLR-4, mediating an inflammatory reaction; (2) in the second theory, HA does not bind to TLRs, but is capable of regulating the interactions of TLRs with their ligands through a pericellular gelatinous barrier.^58^

Ferkel et al.^59^ conducted a systematic review of 40 studies, including 20 preclinical and 20 clinical investigations of intra-articular HA in knee OA, and identified molecular weight (*M*_W_) as the primary determinant of therapeutic performance. Preparations of high *M*_W_ HA (>1 MDa) consistently achieved pain reduction exceeding the minimal clinically important difference (MCID) in randomized trials, whereas lower-*M*_W_ formulations (<500 kDa) failed to reach that threshold. The authors propose that the larger size and prolonged synovial residence of HMW-HA enhance its steric occupation of surface receptors such as CD44 and possibly Toll-like receptors, thereby potentiating anti-inflammatory and chondroprotective effects relative to LMW-HA.

Layilin (LAYN) is a 55 kDa type I transmembrane protein encoded on chromosome 11 that shares homology with C-type lectins. It was identified as a bona fide receptor for HA, mediating direct binding to HA oligosaccharides and, upon engagement, clustering in membrane ruffles and focal adhesion-like sites where it colocalizes with actin-binding proteins to link the HA network to the cytoskeleton, thereby orchestrating cell adhesion, motility, and migration. LAYN expression is upregulated in various cell types, particularly epithelial and immune cells within tumor microenvironments, and transduces extracellular HA cues into intracellular signaling events that regulate focal adhesion dynamics, cell contacts, and broader processes of tissue remodeling, development, homeostasis, and disease.^60^

Intercellular adhesion molecule-1 (ICAM-1) serves as a functional cell surface receptor for HA in inflammatory conditions, including OA. HMW-HA binds to ICAM-1 on synovial cells, mediating endocytic uptake and suppressing the activation of factor nuclear kappa B (NF-κB) and PI3K/Akt pathways, which leads to a reduction in the production of pro-inflammatory cytokines such as tumor necrosis factor alpha (TNF-α), interleukin-1 beta (IL-1β), and interleukin-6 (IL-6).^44^ Further supporting these findings, studies using U937 macrophage assays revealed that pretreatment with HMW-HA significantly inhibits LPS-induced production of TNF-α, IL-1β, and IL-6. Notably, this anti-inflammatory effect was completely abolished when cells were pretreated with an anti-ICAM-1 antibody, confirming that HA’s signaling and cytokine suppression are ICAM-1-dependent.^61^

The receptor for hyaluronic acid-mediated motility (RHAMM or CD168) exists in multiple isoforms localized to the plasma membrane, cytoplasm, and nucleus.^62^ Intracellular RHAMM associates with cytoskeletal proteins (e.g., microtubules and actin) in both the cytoplasm and nucleus, whereas extracellular RHAMM forms complexes with CD44 at the cell surface to mediate downstream signaling.^61^ The HA-RHAMM complex on the cell surface plays a key role in activating signaling pathways involving the proto-oncogene tyrosine-protein kinase Src and other focal adhesion protein kinase complexes.^18^

In OA cartilage repair, low-molecular-weight HA fragments engage RHAMM/CD168 on infiltrating macrophages, which in OA are skewed toward a pro-inflammatory M1 phenotype. RHAMM expression is upregulated in these pro-inflammatory macrophages, and blocking hyaluronic acid–RHAMM interactions with a 15-amino-acid RHAMM-mimetic peptide combined with high-molecular-weight HA shifts macrophages toward an anti-inflammatory M2 profile, marked by decreased TNF-α and IL-1β and increased Interleukin-10 (IL-10), and dramatically improves full-thickness cartilage defect repair in a rabbit microfracture model.^63^ These findings establish RHAMM as a critical nexus through which HA fragments regulate macrophage polarization and drive effective cartilage regeneration.^55^

The lymphatic vessel endothelial hyaluronic acid receptor 1 (LYVE-1) is a transmembrane glycoprotein bearing a link module homologous to CD44; it is prominently expressed on lymphatic capillary endothelium in skin and other tissues and on a subset of tissue-resident macrophages, where it mediates high-affinity binding and internalization of HA for transport into lymphatics. Its primary physiological role is to clear interstitial HA fragments, thereby regulating tissue hydration and extracellular matrix turnover.^64^ Moreover, LYVE-1 on lymphatic endothelial cells forms coreceptor complexes with fibroblast growth factor 2 (FGF2), facilitating FGF2-dependent lymphangiogenic signaling, and with prostaglandin E_2_ (PGE_2_) via EP2 receptors, modulating junctional permeability and leukocyte trafficking during inflammation and repair.^65,66^

The hyaluronic acid receptor for endocytosis (HARE), also known as stabilin-2, is primarily expressed in endothelial cells, specifically in those of the lymphatic system and blood vessels. HARE is highly expressed in sinusoidal liver cells, whose function is to act in the systemic clearance of HA and other GAGs, including natural and infused heparin, and extracellular matrix components (CS, collagen propeptides, advanced glycation end-products, and small phosphatidylserine-based particles).^67^

### Analysis of Clinical Controversies and Guideline Recommendations

The evidence base for intra-articular HA in OA is markedly heterogeneous. While an early high-level review flagged both limited efficacy and safety concerns,^68^ more recent syntheses have yielded mixed results. A 2024 network meta-analysis of 57 RCTs found intra-articular HA for knee OA clinically equivalent to placebo for pain relief at 12 weeks (SMD = −0.04; 95% CrI −0.19 to 0.11; 11 trials) and associated with higher odds of serious adverse events (odds ratios = 1.86; 95% CrI 1.16–3.03).^69^ By contrast, an umbrella review of 25 systematic reviews and meta-analyses reported that intra-articular HA provides moderate pain relief (SMD = −0.20 to −0.50) and modest functional gains (SMD = −0.25 to −0.40) in early to-moderate knee OA, albeit with wide variation according to formulation and trial quality; most preparations cause only mild local reactions, though a minority demonstrate increased dropout rates, underscoring the conflicting safety profile.^8^

Evidence from high-quality studies shows inconsistent results for intra-articular HA in OA. A meta-analysis of 89 randomized controlled trials (*n* ≈ 12,667) concluded that intra-articular HA provides only a small, clinically irrelevant benefit for pain and is linked to a higher risk of serious adverse events (RR 1.41; 95% CI 1.02–1.97).^70^ A multicenter double-blind RCT (*n* = 337) found that five weekly intra-articular HA injections were no more effective than placebo for pain, function, or paracetamol use over 12 months.^71^ Similarly, another double-blind RCT (*n* = 196) in mild-to-moderate knee OA showed no superiority of three weekly intra-articular HA injections over placebo after 6 months, with both groups improving from baseline.^72^

Given this uncertain efficacy, alternative intra-articular injectables have gained attention. In a 2024 network meta-analysis that included 48 studies (9338 knees), platelet-rich plasma (PRP) had the highest probability of being the best treatment for pain and function (surface under the cumulative ranking curve [SUCRA] = 91.54), followed by bone marrow aspirate concentrate (BMAC; SUCRA = 76.46) and HA (SUCRA = 53.12). Corticosteroids showed a much lower SUCRA (15.18). Although HA outperformed placebo (SUCRA = 13.70), its effectiveness was markedly lower than that of PRP or BMAC.^73^

Despite these mixed data, major guideline bodies carve out a role for intra-articular HA in refractory cases. The Osteoarthritis Research Society International (OARSI) conditionally recommends intra-articular HA for knee OA, citing a favorable long-term safety profile versus corticosteroids and potential symptom improvement at or beyond 12 weeks.^74^ In contrast, the American College of Rheumatology and the American Academy of Orthopedic Surgeons recommend against the routine use of intra-articular HA for knee and hip OA, highlighting the limited efficacy and inconsistent evidence of clinically meaningful benefit.^75,76^ Notably, both OARSI and the U.S. Department of Veterans Affairs/Department of Defense (VA/DoD) guidelines continue to list intra-articular HA as an option for patients with persistent symptoms of knee OA despite adequate trials of nonsteroidal anti-inflammatory drugs.^77^

## Chondroitin Sulfate in Osteoarthritis: Applications and Mechanisms

### Clinical Efficacy and Safety Profile of Oral CS

Evidence on the efficacy and safety of chondroitin sulfate (CS) mainly stems from studies using pharmaceutical-grade preparations (pCS), which are characterized by well-defined purity and consistent oligosaccharide composition.^5^

In line with mechanistic and formulation data, a 2024 systematic review and meta-analysis of 13 randomized, placebo-controlled trials found that oral CS significantly reduced pain intensity, improved physical function, and was well tolerated at doses up to 1200 mg/day without any significant increase in adverse events versus placebo in patients with knee OA.^5^ Together, these findings confirm that pCS not only delivers consistent pharmacological activity but also translates into clinically meaningful symptom relief. However, heterogeneity in CS source, manufacturing processes, daily dose (400 mg vs 800 mg), and trial quality contributes to variable outcomes across studies. Table 3 summarizes key clinical investigations of CS in OA.

**Table 3:** Comparative Summary of Clinical Studies on the Use of Chondroitin Sulfate in Osteoarthritis (Predominantly Knee)^a^

study type	dosage	objective	sample	clinical outcomes	nutraceutical presentation
systematic review and network meta-analysis of RCTs	1200 mg/day	to compare the 6 month efficacy of various pharmacological interventions on pain and physical function in knee OA	80 RCTs	pCS vs placebo: significant pain reduction (SMD = −0.26, 95% CrI −0.44 to −0.08); modest improvement in function; ranked among top SYSADOAs for pain relief^78^	oral capsules (400 mg each; 3 capsules)
literature review	800 mg/day (hpCS) and 1200 mg/day (oral gel)	evaluate efficacy, safety, and pharmacoeconomic benefits	clinical data from OA studies in multiple joints	CS improved pain and function with good tolerability^79^	pharmaceutical-grade CS formulations in pills and oral gel
RCT	800 mg/day	define a responder profile for pharmaceutical-grade CS in knee OA	199 patients	significant visual analog scale pain reduction and Lequesne function improvement, especially when treatment <5 years of diagnosis^80^	capsules (400 mg, 2× daily)
meta-analysis of 8 controlled clinical studies	100 mg i.m. (1 mL)	assess the efficacy and safety of Chondroguard as an adjunct in OA therapy	771 OA patients	significant pain reduction and functional improvement; safety similar to control^81^	injectable solution (100 mg/mL) i.m. ×3 injections, (200 mg/2 mL) i.m. every other day
critical review of the literature	1200 mg/day	critically analyze biological effects, clinical efficacy, and quality of chondroitin sulfate supplements in OA	RCTs and original studies	pain reduction and functional improvement, with prolonged effects^82^	sachet of powder for dissolution in water (1200 mg/day)
systematic review and meta-analysis of RCTs	1200 mg/day	evaluate the efficacy and safety of GS, CS, and combinations in knee OA	13 RCTs	CS alone showed significant improvement in pain and function^5^	oral capsules (400 mg, 3× daily)
randomized, double-blind, multicenter, 1 year (intermittent)	800 mg/day	evaluate intermittent CS (3 mo × 2) vs placebo on symptoms and structure	120 randomized (intention-to-treat *n* = 110; completers *n* = 84)	algofunctional index and visual analog scale improved more with CS (AFI −36% vs – 23%); reduced paracetamol use; no joint space width change in CS group vs joint space width decrease in placebo at 12 mo; well tolerated^83^	Sachet (800 mg/day)
multicenter RCT, double-blind, placebo-controlled (24 wk + 8 wk FU)	1 g/day	assess efficacy and tolerability on pain and function	307 randomized (153 CS; 154 placebo)	small but significant pain reduction (Δ6.3 mm favoring CS, *p* = 0.029); Lequesne NS; responder rate 68% vs 56% (*p* = 0.03); biomarkers and adverse events similar^84^	capsule (500 mg twice daily)
multicenter, randomized, double-blind, placebo-controlled pilot RCT	800 mg/day	assess the effect on cartilage volume loss, bone-marrow lesions, synovitis, and symptoms using magnetic resonance imaging	69 randomized (62 completed DB phase; 54 completed 12 mo)	less cartilage volume loss vs placebo at 6 and 12 months (global knee, lateral compartment, tibial plateaus); Bone-marrow lesions reduced at 12 months (lateral compartment); symptoms similar between groups; good safety profile^85^	capsules (800 mg/day, two × 400 mg)
multicenter RCT, double-blind, placebo-controlled, 2 years	800 mg/day	assess long-term effects of CS on radiographic progression and symptoms	622 randomized (309 CS; 313 placebo)	↓ joint space width loss (0.07 vs 0.31 mm; *p* < 0.0001), fewer progressors (28% vs 41%; relative risk reduction 33%), faster pain improvement (visual analog scale, Western Ontario and McMaster Universities Osteoarthritis Index pain), good safety^86^	sachets with purified chondroitin 4 & 6 sulfate (800 mg/day)
post hoc cost-effectiveness analysis of RCT	800 mg/day	assess the cost-effectiveness of CS vs placebo over 6 months in knee OA	CS: *N* = 199; placebo: *N* = 205	CS vs placebo yielded a mean ICER of €33,462 per QALY gained (95% CI 5130–61,794), with a 93% probability of cost-effectiveness at a threshold of €91,870/QALY^87^	tablet (800 mg/day)

a RCT—randomized controlled trial; SMD—standardized mean difference; CrI—credible interval; SYSADOA–symptomatic slow-acting drug for osteoarthritis; IM—intramuscular; KBD—Kashin–Beck disease; GS—glucosamine sulfate; CS—chondroitin sulfate; ICER—incremental cost-effectiveness ratio; QALY—quality-adjusted life year.

In a systematic review and meta-analysis of long-term pain management drugs for knee OA, CS prescription significantly improved joint-space narrowing.^88^ There are no known interactions between CS preparations and food, herbs, or supplements. However, daily recommendations with a good safety profile are doses of up to 1200 mg per day.^79^ Gibbs et al.^89^ report that higher-quality clinical practice guidelines, including the 2019 American College of Rheumatology/Arthritis Foundation guideline, recommend against the use of CS for knee and hip OA and advise against combination products containing GlcN and CS.

The European Society for Clinical and Economic Aspects of Osteoporosis and Osteoarthritis (ESCEO) recommends pCS as a long-term background in Step 1 therapy, as an alternative to prescription-grade crystalline glucosamine sulfate (pCGS) for knee OA. Among the recommended SYSADOA, CS has the greatest evidence (strong recommendation) in terms of therapeutic efficacy; however, this prescribed form must be differentiated from over-the-counter products (over-the-counter medications). ESCEO provides weak recommendations for the combined use of CS and GlcN.^90^

The American College of Rheumatology/Arthritis Foundation only conditionally recommends the use of CS in patients with hand OA.^78^ The Russian Ministry of Health has incorporated parenteral CS into its clinical guidelines for managing various conditions, including “Chronic Pain in Elderly and Senile Patients” (2020), “Falls in Elderly and Senile Patients” (2020), and “Knee Osteoarthritis” (2021), with a grade A recommendation indicating strong confidence in efficacy and safety.^91^

### Pharmacokinetics and the Impact of Formulation

The oral bioavailability and pharmacokinetic profile of CS are complex and critically influenced by its molecular weight, source, and formulation. Preparations sourced from bovine, porcine, avian, or marine origins differ markedly in average molecular weight (ranging from ≈10–40 kDa to 50–100 kDa) and sulfation patterns, which directly impact their gastrointestinal processing and absorption.^92,93^ Upon oral administration, high-molecular-weight (HMW) CS polymers are poorly absorbed intact due to their hydrophilic nature and large size. The absolute bioavailability of these native polymers is generally low, estimated to be below 15%. The majority of an oral dose undergoes extensive enzymatic and bacterial catabolism in the colon, where gut microbiota degrades them into lower-molecular-weight sulfated oligosaccharides and disaccharides (e.g., ΔUA-GalNAc-4S/6S). These smaller, more absorbable fragments are then transported into the systemic circulation.^94,95^

Pharmacokinetic studies in humans after a single dose of 800–1200 mg of pharmaceutical-grade CS reveal a profile characterized by slow absorption and a long elimination phase. Peak plasma concentrations (*C*_max_) of CS-derived disaccharides are typically low, reaching only nanomolar to low micromolar ranges (e.g., 10–50 ng/mL), and occur at a time to peak (*T*_max_) of approximately 4 to 8 h postadministration.^95,96^ The elimination half-life (t_1_/_2_) is relatively prolonged, estimated between 5 and 15 h, suggesting sustained release from intestinal depots or connective tissues and aligning with its proposed once daily dosing regimen. This pharmacokinetic behavior, with a low peak concentration but sustained presence, is thought to be crucial for its cumulative anti-inflammatory and chondroprotective effects, rather than immediate high systemic exposure.^96^

The critical role of molecular weight on absorption was unequivocally demonstrated in a 2023 study, where the administration of CS oligosaccharides (CS-Oligos) with molecular weights below 3 kDa resulted in significantly higher plasma concentrations and urinary excretion rates in humans compared to standard HMW-CS (70 kDa). This confirms that strategies aimed at reducing molecular size, such as the production of low-molecular-weight CS (LMW-CS) or specific oligosaccharides, can significantly enhance intestinal absorption and systemic bioavailability, potentially addressing the variability in clinical efficacy observed with traditional preparations.^97^

Despite absorption, the direct penetration of CS or its metabolites into chondrocytes is limited. Therefore, its primary anti-inflammatory and modulatory effects are largely attributed to the interaction of these circulating fragments with membrane receptors on chondrocytes, synoviocytes, and immune cells (e.g., CD44, TLR4, ICAM-1), modulating intracellular signaling pathways and the inflammatory response.^92^

### Mechanisms of Action: Receptor-Mediated Modulation of Inflammation

CS can mediate various biological effects through its interaction with specific receptors. After oral or intra-articular administration, CS reaches the synovial joint, where it interacts with membrane receptors expressed on chondrocytes, synoviocytes, osteoblasts, and macrophages, including CD44, ICAM-1, RHAMM, TLR-2/4, and HARE (Table 4). These interactions facilitate its internalization or trigger intracellular signaling cascades that modulate inflammation and cartilage metabolism.

**Table 4:** Summary of Receptors Involved in Chondroitin Sulfate Functions^a^

receptors	location	mechanism
lectin receptors HARE	cell surface	lectins recognize membrane-bound glycoconjugates and are subsequently endocytosed, degraded, and involved in cellular functions^101^
TLR-2/4 and CD44	cell surface	they mediate CS internalization and trigger anti-inflammatory responses^92^
RHAMM, ICAM-1, and RPTPσ	cell surface	bind chondroitin sulfate to (i) promote cell migration and tissue repair via ERK1/2 activation and cytoskeletal remodeling; (ii) enhance leukocyte adhesion through β2-integrins; and (iii) modulate immune activation and cytokine production through tyrosine de/phosphorylation of signaling substrates^102^
NgR1 and NgR3	highly expressed in the brain and liver	implicated in neuronal inhibition, binds with high affinity to the glycosaminoglycan portion of proteoglycans, and participates in CSPG inhibition in cultured neurons^103^
Annexin 6	in the cytoplasm, it also exists on the cell surface	implicated in many biological processes, including cell proliferation, survival, differentiation, inflammation, and viral infection^104^

a Lectin receptors HARE—hyaluronic acid receptor for endocytosis; TLR-2/4—toll-like receptors 2 and 4; CD44—cluster of differentiation 44; RHAMM—receptor for hyaluronic acid-mediated motility; ICAM1—intercellular adhesion molecule 1; NgR1 and NgR3—Nogo receptors 1 and 3, família NgR; RPTPσ—receptor-type sigma protein tyrosine phosphatase.

In human chondrocytes stimulated with IL-1β, treatment with highly purified chondroitin sulfate (hpCS) reduces the phosphorylation of p38 mitogen-activated protein kinase (p38MAPK) and extracellular signal-regulated kinase 1/2 (ERK1/2), inhibits the nuclear translocation of NF-κB, and decreases nitric oxide synthase 2 (NOS-2) activity, resulting in reduced nitric oxide (NO) production in the joint environment.^79^ CS also suppresses the production of other pro-inflammatory mediators, such as TNF-α, IL-6, IL-1β, and Prostaglandin E_2_ (PGE2), in both primary human chondrocytes and macrophages (Figure 3).^98,99^

**Figure 3:**
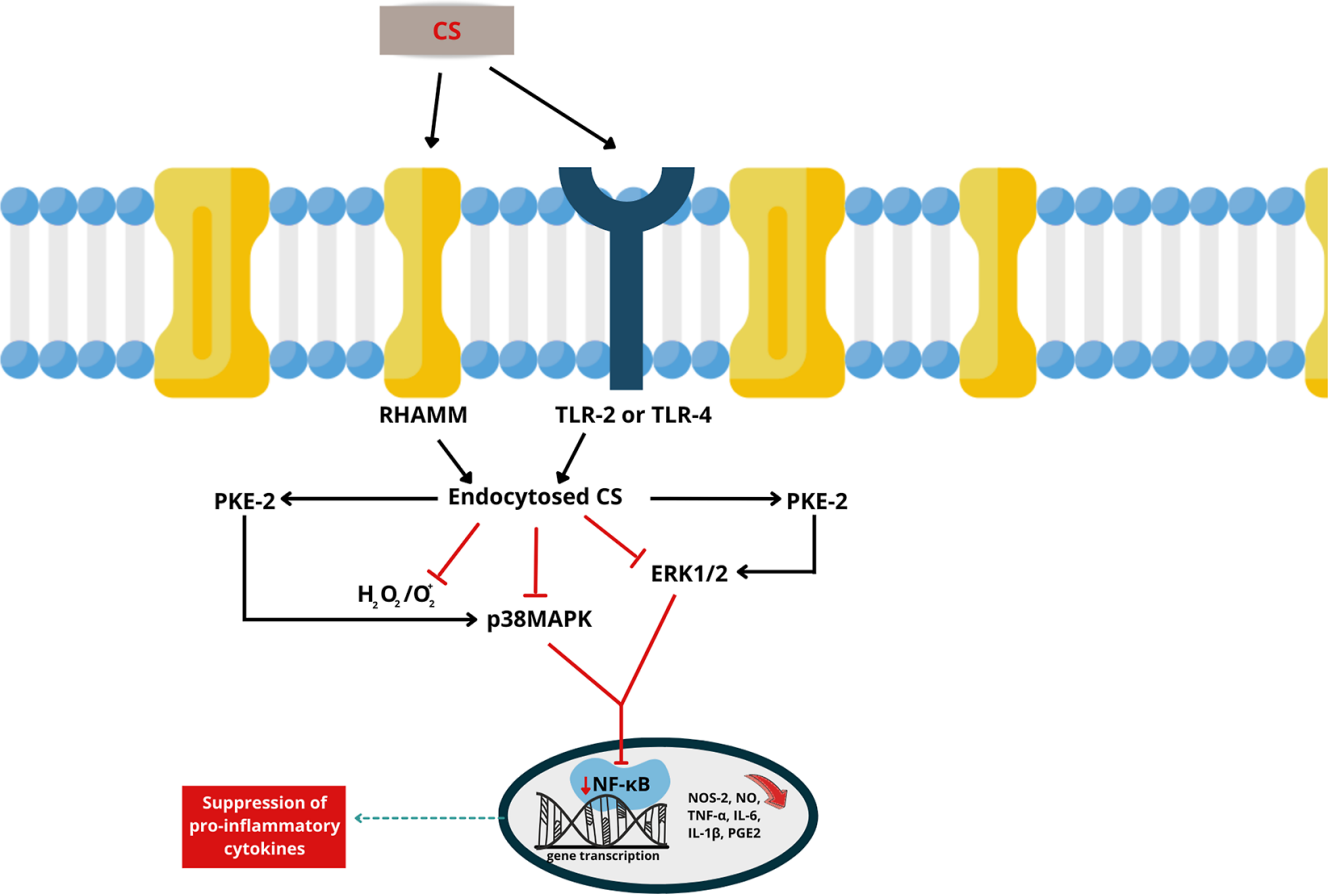
OA inflammatory pathways and anti-inflammatory activity of CS. CS: chondroitin sulfate. RHAMM: receptor for hyaluronic acid-mediated motility. TLR-2 or TLR-4: toll-like receptor 2 or 4. PKE-2: pyruvate kinase isoenzyme type 2. H_2_O_2_: hydrogen peroxide. O_2_^–^: superoxide anion. p38MAPK: p38 mitogen-activated protein kinase. ERK1/2: extracellular signal-regulated kinase 1/2. NF-κB: nuclear factor kappa-light-chain-enhancer of activated B cells. NOS-2: nitric oxide synthase 2. NO: nitric oxide. TNF-α: tumor necrosis factor alpha. IL-6: interleukin-6. IL-1β: interleukin-1 beta. PGE2: prostaglandin E2.

The anti-inflammatory activity of CS varies depending on its source and sulfation pattern. CS-4S and CS-6S, derived from bovine trachea, porcine trachea, ray cartilage, and chicken sternum, show different efficiencies in suppressing cytokine and NO production in RAW 264.7 cells and primary cultures. For example, in RAW macrophages, CS significantly reduces TNF-α and IL-1β levels, while the effect on IL-6 is less pronounced.^98^

In vitro, CS demonstrates beneficial effects on the metabolism of chondrocytes, synoviocytes, macrophages, and subchondral bone cells.^98,99^ Its exogenous administration in OA models modulates cell signaling pathways and promotes anti-inflammatory, antioxidant, antiapoptotic, anticatabolic, and anabolic effects.^17^ In LPS-induced bone marrow-derived macrophages, CS-4S, CS-6S, and CS from bovine and porcine trachea reduce the release of TNF-α, IL-6, IL-1β, and NO, highlighting distinct responses between RAW and primary macrophage models.^100^

An in vitro model of OA stimulated with hydrogen peroxide (H_2_O_2_) to induce apoptosis in primary chondrocyte culture when challenged with CS from sturgeon bone (composed of chondroitin-4-sulfate and chondroitin-6-sulfate), increased cell viability, reduced the rate of deoxyribonucleic acid (DNA) fragmentation and apoptosis, as well as protected mitochondria and reduced caspase-3 and caspase-9 expression.^105^ These results suggest that sturgeon bone CS improves cartilage matrix damage by inhibiting chondrocyte apoptosis. During the progression of OA, chondrocyte apoptosis is associated with several pathological events, including the reduction of chondrocyte numbers, decreased extracellular matrix (ECM) synthesis, altered gene expression with the production of pro-inflammatory cytokines (such as interleukins IL-1β, IL-6, TNF-α), and increased alkaline phosphatase (ALP), resulting in abnormal calcification of the subchondral bone.^106^

Chondroitin sulfate from sturgeon bone (CSSB) was evaluated in an in vitro model of oxidative injury in primary rat chondrocytes induced by hydrogen peroxide (H_2_O_2_). CSSB at moderate (200 μg/mL) and high (400 μg/mL) doses significantly enhanced cell viability (CCK 8 assay) and reduced the apoptotic rate (Annexin V PI flow cytometry) compared to the H_2_O_2_ model group. Concomitantly, CSSB treatment markedly decreased the levels of pro-inflammatory cytokines interleukin (IL-6), IL-8, and interferon-gamma (IFN-γ) in the culture supernatant (ELISA). Finally, Western blot analysis showed that CSSB restored the expression of Wnt β-catenin pathway proteins: Wnt3a, Frizzled5, dishevelled, β-catenin, and c-Myc while attenuating the elevated phosphorylation of GSK 3β, indicating modulation of survival and inflammatory signaling cascades.^107^

A study demonstrated that a collagen scaffold functionalized with isolated CS blocks LPS binding to CD44 and inhibits NF-κB activation in bone marrow—derived macrophages under LPS stimulation, resulting in significant decreases in pro-inflammatory mediators, including IL-6, TNF-α, IL-1β, inducible nitric oxide synthase, and prostaglandin E_2_ synthase.^102^ The anti-inflammatory and chondroprotective effects observed in clinical settings are supported by mechanistic data from animal models. For instance, Ma et al.^108^ administered isolated CS systemically in an ACL-transection rat model of OA and observed marked inhibition of Inhibitor of kappa B alpha (IκBα) and p65 subunit of the NF-κB complex (p65) phosphorylation, reduced levels of TNF-α, IL-6, and IL-1β in synovium and cartilage, and preservation of type II collagen and aggrecan, collectively attenuating extracellular matrix degradation in vivo. This preclinical evidence provides a mechanistic foundation for the modulation of NF-κB signaling observed in human studies.

Golovach et al.^17^ reviewed the molecular mechanisms by which CS modulates inflammatory processes in post-traumatic OA. In this review, the authors highlight that CS reduces activation of the NF-κB factor transcription and p38 MAPK, while also promoting activation of the redox regulator Nrf2; in in vitro models of LPS-treated chondrocytes, CS likewise inhibits IL-1β secretion and decreases nuclear translocation of the canonical NF-κB subunits (p65 and p50), suggesting an action on NF-κB pathway activation.

Chondroitin sulfate (500 mg/kg/day, orally for 8 weeks) in streptozotocin (STZ)-induced diabetic osteoporosis rats significantly lowered fasting blood glucose and improved bone mineral density in the femur and lumbar vertebrae. It restored bone morphology, normalized histomorphometric parameters, and reduced the number of femoral osteoclasts and tibial adipocytes. Serum levels of IL-1β, IL-6, and TNF-α were markedly decreased, while the activities of superoxide dismutase (SOD), glutathione peroxidase (GPX), and catalase (CAT) increased, alongside elevated malondialdehyde (MDA), indicating a combined anti-inflammatory and antioxidative effect. The treatment also downregulated alkaline phosphatase (ALP), C-terminal telopeptide of type I collagen (CTX-1), tartrate-resistant acid phosphatase 5b (TRACP 5b), osteocalcin, and receptor activator of nuclear factor-κB ligand (RANKL), and upregulated runt-related transcription factor 2 (RUNX2) and osteoprotegerin (OPG), shifting the OPG/RANKL axis toward bone preservation. These coordinated actions confirm chondroitin sulfate’s potential to prevent inflammation-driven bone degradation relevant to OA.^109^

Chang et al.^98^ explored the therapeutic potential of chondroitin sulfate oligosaccharides (oligo-CS) in mitigating OA by targeting the NLRP3 inflammasome. Their study demonstrated that OCP (octacalcium phosphate) crystals trigger NLRP3 activation, NF-κB signaling, and the release of inflammatory cytokines (IL-1β, IL-6), leading to cartilage matrix degradation via MMP-13 and disintegrin and metalloproteinase with thrombospondin motifs 5 (ADAMTS-5). Oligo-CS treatment significantly reduced these inflammatory responses, preserved cartilage integrity, and inhibited catabolic markers. In vivo, oral administration of oligo-CS in mice protected joint structure and restored gut microbiota balance. These findings showed that oligo-CS could be a promising candidate for OA treatment by addressing inflammation and cartilage degradation.

### Analysis of Clinical Controversies and Guideline Recommendations

Current evidence indicates that pharmaceutical-grade chondroitin sulfate (pCS) is generally well tolerated in OA patients, with treatment-emergent adverse events as rare as with placebo and less frequent than with most NSAIDs; in the CONCEPT trial, 800 mg/day of pCS demonstrated a safety profile equivalent to celecoxib over six months without any significant increase in serious adverse events.^80^ Network meta-analyses further confirm that celecoxib carries the highest odds of adverse events, whereas pCS, alone or combined with glucosamine, tends to produce fewer and generally mild side effects.^110^

However, efficacy findings remain inconsistent: a 2023 meta-analysis of 13 randomized controlled trials reported modest but statistically significant reductions in pain and improvements in function for knee OA treated with pCS versus placebo,^111^ whereas a 2024 critical review concluded that pCS confer minimal clinically meaningful benefit on joint space narrowing or pain beyond 12 months of follow-up.^82^ The root of this contradiction can be traced back to a landmark 2007 meta-analysis by Reichenbach et al., which pooled 20 randomized trials (*n* ≈ 3846 patients). This study highlighted high heterogeneity and indications of publication bias. Most importantly, when the analysis was restricted to large-scale trials with intention-to-treat analysis, the symptomatic effect of chondroitin sulfate virtually disappeared (effect size ≈0.03; 95% CI – 0.13 to 0.07), corresponding to a clinically irrelevant difference on a 100 mm VAS (≈0.6 mm). Evaluations of radiographic outcomes showed minimal gains (tenths of a millimeter), leading the authors to conclude that the symptomatic benefits of chondroitin sulfate are negligible or nonexistent and to recommend against its routine use in clinical practice.^112^

Moreover, earlier network meta-analyses found that neither pCS alone nor glucosamine plus CS combinations achieved clinically important pain relief at one year.^110^ A large randomized, placebo-controlled trial including 1583 patients with knee OA found that daily chondroitin sulfate for 24 weeks did not significantly reduce pain in the overall population. However, some benefit was observed in patients with moderate to severe baseline pain.^113^ A network meta-analysis of 10 large-scale randomized trials involving 3803 patients with hip or knee OA found that CS did not provide clinically important improvements in joint pain or radiographic progression compared with placebo.^114^

Reflecting these mixed results, the American College of Rheumatology guideline of 2019 and the American Academy of Orthopedic Surgeons guideline of 2021 both strongly recommend against the use of CS, alone or in combination with GlcN, for knee and hip OA, citing the low quality of evidence and lack of clinically relevant benefit.^75,76^ Similarly, the 2020 OARSI update advises against its use in nonsurgical knee OA management.^115^ Finally, pharmacokinetic considerations may further complicate the interpretation of combination therapies. Conrozier and Lohse^116^ reported that coadministration of GlcN with CS reduces GlcN absorption by approximately 58%, potentially diminishing combined efficacy.

## Glucosamine Sulfate in Osteoarthritis: Applications and Mechanisms

### Clinical Efficacy and Safety Profile of Oral GlcN

Glucosamine (GlcN; 2-amino-2-deoxy-β-d-glucopyranose), an amino sugar with a molecular weight of 197.17 g/mol (PubChem, 2024), plays a key role in the biosynthesis of proteoglycans, which are essential for maintaining the integrity and function of articular cartilage.^117,118^ Clinical studies have demonstrated that GS can positively impact cartilage structure, improve physical function, and reduce pain in individuals with OA (Table 5).

**Table 5:** Comparative Summary of Clinical Studies on the Use of Glucosamine in Osteoarthritis (Predominantly Knee)^a^

study type	dosage	objective	sample	clinical outcomes	nutraceutical presentation
systematic review	1500 mg/day	map and classify various health outcomes associated with glucosamine sulfate use, evaluating its efficacy and safety in OA patients	37 RCTs were included in the analysis	GS was more effective than placebo in 9 out of 17 outcomes, including improvements in joint space, OA progression, glycemic control, and physical performance^125^	oral capsules (750 mg, 2 × daily)
RCT	1500 mg/day	evaluate the synergistic effect of high-power laser therapy (HPLT) combined with pCGS on knee OA	90 patients	significant pain reduction and improved function at 6 months (T2) in the HPLT + pCGS group compared to HPLT + placebo^126^	oral solution (1500 mg pCGS)
retrospective cohort	1500 mg/day	to compare changes in objective functional performance (5× SST, TUGT, 3MWDT) from baseline through 1 year between oral pCGS and PRP in knee OA patients	204 patients received pCGS and 102 received PRP	TUGT improved by 0.5 s at 6 weeks and 1.2 s at 1 year; 5× SST improved by 1.7 s at 12 weeks and 2.5 s at 1 year; 3MWDT increased by 6.8 m at 12 weeks and 7.7 m at 1 year^119^	oral capsules (500 mg pCGS, 3× daily)
open-label prospective study	1500 mg/day	evaluate the efficacy of CGS for knee OA compared to NSAIDs	111 patients (both sexes) with knee OA (52 treated with CGS)	after 12 months, both groups had reduced pain intensity, but CGS showed a significantly greater reduction compared to NSAIDs^120^	sachet (1500 mg CGS powder)
RCT	750 mg/day	assess glucosamine efficacy for selected OA symptoms in elderly nursing home residents	60 participants were randomly assigned to glucosamine (*n* = 30) vs control	glucosamine therapy significantly reduced joint pain and OA symptoms, with anabolic cartilage benefits^127^	oral capsules (750 mg, 2× daily)
systematic review based on RCTs	1500 mg/day	evaluate the efficacy and safety of glucosamine in knee OA	15 RCTs of knee OA patients	significant pain reduction, improvement in physical function, and no significant adverse events^117^	capsules or tablets (500 mg GS, 3× daily)
systematic review	1500 mg/day	critically assess the efficacy of oral glucosamine for temporomandibular joint OA	8 RCTs of adults with temporomandibular joint OA	significant pain reduction in TMJ, increased mouth opening, and lasting anti-inflammatory effects^128^	tablets (500 mg, 3× daily)
RCT	1500 mg/day	to test whether long-term glucosamine sulfate modifies structural progression and symptoms in knee OA	202 patients	slowed structural progression: joint space width change W0 → 3 years placebo −0.19 mm vs glucosamine +0.04 mm (*p* = 0.001); fewer severe narrowings (5% vs 14%); symptoms improved ∼15–25% (Lequesne/Western Ontario and McMaster Universities Osteoarthritis Index); safe^129^	sachet (1500 mg pCGS powder)
RCT	1500 mg/day	to assess long-term structural and symptomatic effects of GS	212 patients	GS prevented joint space narrowing (joint space width) vs significant joint space width loss in the placebo^130^	sachet (1500 mg pCGS powder)
systematic review and meta-analysis	1500 mg/day	assess the exclusive effects of GS on knee OA	25 RCTs included: 9 exclusively for GS	significant reduction in tibiofemoral joint-space narrowing^5^	capsules or tablets (500 mg GS, 3× daily)

a GS—glucosamine sulfate; RCT—randomized controlled trial; HPLT—high-power laser therapy; pCGS—pharmaceutical crystalline glucosamine sulfate; PRP—platelet-rich plasma; TUGT—timed up and go; 5× SST—five times sit-to-stand test; 3MWDT—three-minute walk test; CGS—crystallized glucosamine sulfate; NSAIDs—non-steroidal anti-inflammatory drugs; TMJ—temporomandibular joint.

Recently, a propensity-score-matched study compared patented crystalline glucosamine sulfate (pCGS) with PRP injections, and results showed that after 12 weeks, with effects sustained at 12 months, patients receiving pCGS exhibited significant improvements in the five-times sit-to-stand test (5× SST), timed up-and-go test (TUGT), and 3 min walk test (3MWDT) at the same 1500 mg daily dose.^119^ Although some long-term studies have not demonstrated consistent structural changes, most confirm the maintenance of symptomatic benefits without an increase in adverse events, reinforcing the ongoing safety and efficacy of GS in OA.^120^

A systematic review has shown that standard oral doses of GS (1500 mg/day), alone or combined with CS, did not produce clinically significant changes in Hemoglobin A_1_c (HbA_1_c) or other glucose-monitoring parameters in healthy, overweight/obese, or OA populations.^121^ In a UK Biobank cohort of 404,508 participants with OA, glucosamine sulfate use was associated with a lower incidence of type 2 diabetes and cardiovascular diseases after eight years of follow-up,^122^ and was also linked to decreased overall and cause-specific mortality, including cardiovascular disease, cancer, respiratory disease, and digestive disease, over an average follow-up of 8 years and 9 months.^123^ These protective effects have been attributed to the anti-inflammatory properties of glucosamine, although the underlying molecular basis remains unclear.^124^

A six-month retrospective observational study in 123 patients with erosive hand OA found that adding 1500 mg/day of prescription-grade crystalline glucosamine sulfate (pCGS) to conventional therapy significantly improved clinical outcomes. Compared to conventional therapy alone, the pCGS group showed greater reductions in hand pain (analog visual scale −21.8 mm vs −10.3 mm; *p* < 0.01), improved hand function (functional index for hand OA −4.16 vs −1.57; *p* < 0.001), shorter morning stiffness, better health assessment questionnaire and 36-item Short Form Health Survey scores, and reduced use of paracetamol and NSAIDs (*p* < 0.001), with no serious adverse events reported.^131^ Based on this and other evidence, the ESCEO guidelines classify SYSADOAs, including prescription-grade pCGS, as first-line therapy for symptomatic knee OA, emphasizing that pharmaceutical-grade pCGS offers superior structural and symptomatic benefits compared to over-the-counter glucosamine formulations.^132^ In contrast, the American College of Rheumatology/Arthritis Foundation guideline recommends against the use of glucosamine for knee and hip OA. This position likely reflects the overall evidence for widely available over-the-counter supplements, as the guideline does not distinguish between these and specific prescription-grade formulations.^75^

### Pharmacokinetics and the Impact of Formulation

GlcN preparations are extracted from chitin (crustacean bark) by acid hydrolysis or are chemically synthesized in the laboratory. Being a weak organic base, GlcN must be stabilized as a salt to ensure stability. The three most common forms on the market are glucosamine hydrochloride (GH, from crab shells), glucosamine sulfate (GS, from shrimp shells), and *N*-acetyl glucosamine (GlcNAc, chemically synthesized).^133^ Recommended daily dosages typically range from 1250 to 1500 mg.^117,121^

Despite widespread use, oral glucosamine sulfate shows low mean bioavailability (approximately 25%) and marked interindividual variability, with differences of up to 100-fold, influenced by factors such as fasting and circadian rhythm.^116^ In healthy volunteers receiving 1500 mg/day for 6 days, the time to reach steady-state maximum plasma concentration (*C*_max_) ranged between 1 and 6 h. The coefficient of variation for pharmacokinetic parameters such as minimum plasma concentration at steady state (Css_min_), time to reach maximum concentration at steady state (Tss_max_), and area under the plasma concentration–time curve at steady state (AUC_ss_) exceeded 20%, quantitatively confirming the inconsistency in absorption and elimination.^134^ In 18 healthy volunteers who received a single 1500 mg oral dose of glucosamine sulfate 2KCl, plasma levels ranged from 0.1 to 10 μg/mL, with the lower limit of quantification set at 0.1 μg/mL. Mean peak concentrations were 1010 ng/mL for the chitosan-derived formulation and 1070 ng/mL for the biofermented formulation, reached at 2.0 and 1.6 h postdose, respectively. Total systemic exposure over time, expressed as the area under the plasma concentration–time curve from zero to infinity, was 3940 ng·h/mL for the chitosan-derived product and 4000 ng·h/mL for the biofermented product.^135^

Integrated metabolomic and microbiome studies reveal that oral glucosamine undergoes extensive presystemic metabolism by gut microbiota. Over 50% of the ingested dose is degraded before absorption, and only approximately 10–12% reaches the systemic circulation.^136^ This limited systemic availability results from both microbial degradation and first-pass metabolism in the intestinal epithelium. These processes vary significantly among individuals due to differences in gut microbiota composition and intestinal transporter activity.

Fecal excretion of glucosamine-derived metabolites accounts for about 11.3%, while less than 2% of the compound is excreted unchanged in the urine. Mechanistic studies using Caco-2 cell models demonstrate that glucosamine undergoes intracellular phosphorylation to form glucosamine-6-phosphate, which governs its subsequent metabolic fate. About 70–80% is used intracellularly, and 20–30% is transported transcellularly.^137^

It is crucial to differentiate patented crystalline glucosamine sulfate (pCGS) from other glucosamine sulfate formulations (CGS or over-the-counter GS supplements). pCGS is a pharmaceutical-grade product stabilized with sodium chloride and administered at 1500 mg once daily, producing consistent plasma concentrations near 8–10 μmol/L that are sufficient to inhibit IL-1—induced catabolic gene expression. These pharmacokinetic properties translate into reproducible symptomatic and structural benefits in clinical trials. In contrast, generic CGS and over-the-counter GS supplements are heterogeneous regarding molecular form, composition, stability, and absorption, resulting in unpredictable plasma levels and inconsistent clinical outcomes. Distinguishing pCGS from nonpharmaceutical glucosamine formulations is therefore essential for accurate interpretation of pharmacokinetic data, efficacy, and safety.^138^

### Mechanisms of Action: Receptor-Mediated Modulation of Inflammation

GlcN is a natural constituent of the glycosaminoglycans (GAGs) in cartilage and synovial fluid When administered orally, it penetrates synoviocytes and chondrocytes via glucose transporter (GLUT) transporters (Figure 4), is rapidly O-GlcNAcylated, and initiates an intracellular anti-inflammatory program.^131^ The elevation of O-GlcNAc prevents the phosphorylation of IκBα, blocking the nuclear translocation of NF-κB.^139^ Several mechanisms contributing to the GlcN-induced decline of NF-κB were reported.^140^ Decreased NF-κB activation increases the promoter methylation of pro-inflammatory genes, leading to their reduced transcription.^16^ GlcN stimulates the synthesis of hyaluronic acid and the glycosaminoglycans chondroitin-4-sulfate (C4S) and chondroitin-6-sulfate (C6S), enhancing its anti-inflammatory effects in osteoarthritis. Moreover, because it is taken up via GLUT transporters present in the membranes of synoviocytes and chondrocytes, GlcN penetrates these cells to inhibit NF-κB activation and reduce metalloproteinase (MMP) activity, thereby attenuating the inflammatory and catabolic processes in cartilage.^141^

**Figure 4:**
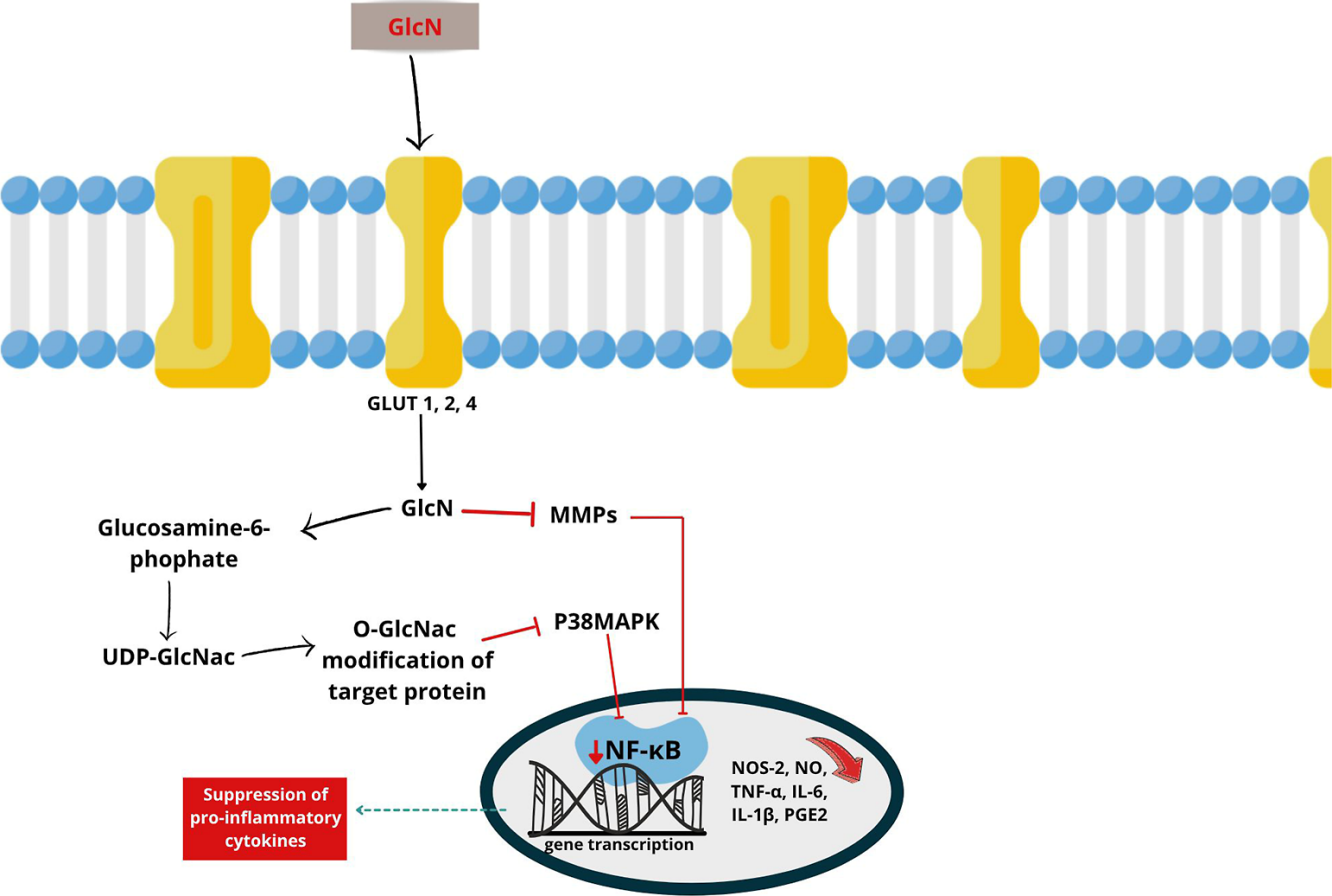
OA inflammatory pathways and anti-inflammatory activity of GlcN. GlcN: glucosamine. GlcN: glucosamine. GLUT 1, 2, 4: glucose transporter 1, 2, 4. MMPs: matrix metalloproteinases. GlcNAc: *N*-acetylglucosamine. UDP-GlcNAc: uridine diphosphate *N*-acetylglucosamine. O-GlcNAc: O-linked β-*N*-acetylglucosamine. P38MAPK: p38 mitogen-activated protein kinase. NF-κB: nuclear factor kappa-light-chain-enhancer of activated B cells. NOS-2: nitric oxide synthase 2. NO: nitric oxide. TNF-α: tumor necrosis factor alpha. IL-6: interleukin-6. IL-1β: interleukin-1 beta. PGE2: prostaglandin E2.

Studies have reported that GlcN can reduce the expression of MMPs in chondrocytes and osteosarcoma cell lines, while increasing the expression of type II collagen (COL2A1) and sirtuin 1 (SIRT1) protein, a regulatory gene of COL2A1.^142^ COL2A1 is the main component of the cartilage matrix, and its degradation and decrease can result in the progression of OA, accelerating chondrocyte hypertrophy in the pathophysiological process of OA.^143^ Evidence indicates that oral GlcN hydrochloride suppressed the nucleotide-binding domain, leucine-rich-containing family, pyrin domain-containing-3 (NLRP3) inflammasome and the IL-1β precursor, contributing to reduced reactive oxygen species (ROS) production and NF-κB activation in mouse and human macrophages activated by LPS. In addition, GlcN interfered with the binding of double-stranded RNA-activated protein kinase (PKR) and protein kinase 7 (NEK7), which are involved in activating NLRP3 and forming the NLRP3/apoptosis-associated speck-like protein containing a caspase recruitment domain (ASC) complex.

GlcN induces autophagy in osteoarthritic chondrocytes by inhibiting mechanistic target of rapamycin (mTOR) through forkhead box O3a (FOXO3a) activation. This effect promotes cytoprotection in the short term but may become detrimental with prolonged exposure. Moreover, in vitro studies using primary human chondrocytes have confirmed that glucosamine enhances autophagosome formation by increasing the levels of microtubule-associated proteins 1*A*/1B-light chain 3-II (LC3-II) and autophagy-related protein (Beclin-1), which are established markers of autophagy. These findings reinforce the therapeutic potential of glucosamine in preserving cartilage homeostasis during the early stages of OA.^144^ In human chondrocytes, when GlcN is exposed for a short-term (2 h), it activates autophagy; however, when exposed for a long time (24 h), it alters the autophagic response and induces apoptosis.^118^ In MC3T3-E1 osteoblasts subjected to H_2_O_2_-induced oxidative stress, GlcN attenuated cytotoxicity by decreasing ROS production and MDA levels while restoring glutathione (GSH) and nitric oxide (NO) levels. It also induced protective autophagy, as evidenced by increased Beclin-1 expression, a higher LC3 II/I ratio, and enhanced autophagosome formation, resulting from the inhibition of protein kinase B (Akt) and mTOR phosphorylation. Activation of Akt with small-molecule AKT activator (SC79) reversed the autophagy effect, confirming the role of the ROS/Akt/mTOR pathway in glucosamine-mediated protection of osteoblasts.^145^

The unfolded protein response (UPR) signaling pathway plays a crucial role in chondrocyte maturation, osteoblast function, and the pathogenesis of osteoarthritis. In OA models, ER stress can be cytoprotective by activating autophagy through the chaperone glucose-regulated protein 78 (GRP78; also known as BiP, gene HSPA5), or cytotoxic by triggering apoptosis via C/EBP homologous protein (CHOP), depending on the intensity and duration of the stress. These opposing outcomes are coordinated by the IRE1/mTORC1/PERK axis (IRE1 = inositol-requiring enzyme 1; mTORC1 = mechanistic target of rapamycin complex 1; PERK = PKR-like ER kinase), which finely tunes the balance between autophagy and CHOP-mediated apoptosis to alleviate protein misfolding in osteoarthritic joints.^146^ To date, no studies have investigated the role of glucosamine-induced endoplasmic reticulum stress, defined as the accumulation of misfolded proteins within the ER lumen that activate adaptive unfolded protein response (UPR) signaling, in osteoarthritis. Experimental evidence suggests that the induction of endoplasmic reticulum stress may help delay OA onset; however, its impact on disease progression and prognosis remains to be elucidated.^142^

GlcN and its derivatives can inhibit osteoclastic differentiation and enhance chondrogenic differentiation.^147,148^ The pCGS was investigated in a 2D/3D coculture system with human primary osteoclasts (hOCS) and osteoblasts (hOBs) from OA patients and healthy individuals. Results showed that pCGS treatment positively affected osteoblast activity, while hOCS isolated from OA patients were more sensitive than those from healthy donors. Furthermore, pCGS exhibited anabolic effects on hOCS, both in conventional 2D cell culture and dynamic hOC/hOB 3D coculture.^149^

Several studies using animal models and human models have reported the anti-inflammatory effects of GlcN in OA.^150,151^ GlcN administered at different concentrations significantly reduced the serum expression of pro-inflammatory cytokines such as IL-1β, IL-6, TNF-α, and C-reactive protein levels, while concomitantly upregulating anti-inflammatory cytokines, such as interleukin-2 (IL-2) and IL-10 in animal models of OA.^131^ In a randomized controlled trial, patients with knee osteoarthritis received either celecoxib 200 mg/day alone or in combination with glucosamine sulfate 1500 mg/day for 8 weeks. The combined treatment group showed a significant improvement in redox status, as evidenced by increased serum superoxide dismutase (SOD) activity and reduced malondialdehyde (MDA) levels, compared to celecoxib alone. This confirms the antioxidant benefit of GlcN supplementation.^140^

### Analysis of Clinical Controversies and Guideline Recommendations

Meta-analyses indicate that GlcN has an insignificant effect on OA or only a small impact on pain relief in OA patients.^88,152,153^ A randomized, double-blind, placebo-controlled trial with 80 patients receiving 1500 mg/day of glucosamine sulfate for six months found no significant reduction in pain compared with placebo, reporting only a minimal and clinically irrelevant improvement in knee flexion.^154^ Another study with 98 patients treated with 500 mg of glucosamine three times daily for two months also showed no significant difference in pain reduction versus placebo, with similar rates of adverse events.^155^ In contrast, a large multicenter trial including 1583 patients with knee osteoarthritis concluded that glucosamine hydrochloride (1500 mg/day) was not superior to placebo for overall pain reduction. However, the combination with chondroitin showed benefit in the subgroup of patients with moderate to severe pain.^113^ Similarly, a Cochrane meta-analysis including 20 RCTs with 2570 patients found that, overall, glucosamine did not significantly improve pain or function compared to placebo, although some studies suggested modest benefits in specific formulations.^156^

However, the translation of these mechanistic findings into structural benefits across all species remains challenging. For example, in an experimental randomized controlled trial involving horses, oral administration of CS at a dose of 1000 mg/day combined with GS at the same dosage did not prevent the progression of cartilage lesions, nor did it reduce the continuous increase of the collagen type II degradation biomarker (CTX-II). It also did not improve macroscopic or histopathological scores of the cartilage, nor produce clinically relevant radiographic or ultrasonographic differences in the subchondral bone compared to the control group.^157^ This highlights the complexity of OA pathophysiology and the potential differences in species response, underscoring the necessity of human clinical trials to confirm efficacy. Furthermore, Čeh and Šarabon^158^ demonstrated that patients with knee osteoarthritis treated with GlcN did not exhibit significant improvements in knee pain or physical function when compared to exercise alone. Consequently, both the American College of Rheumatology and the American Academy of Orthopedic Surgeons recommend against the use of GlcN, whether alone or in combination with CS, for the treatment of knee and hip OA, due to the lack of consistent evidence of clinical benefit.^75,76^

### New OA Treatment Perspectives

To date, pharmacotherapy for OA has shown little progression, especially for acetaminophen, NSAIDs (topical and oral), and opioids, which are widely used to relieve OA symptoms. Furthermore, none of these drugs has been shown to modify the OA progression and prevent long-term disability. For this reason, the Food and Drug Administration (FDA) considers OA to be a severe disease with unmet medical needs.^157^ The therapies presented in Table 6 stand out among the new therapeutic approaches for OA.

**Table 6:** New Therapeutic Perspectives for Osteoarthritis^a^

targets	therapy and dose	phase	clinical efficacy	safety
pain	Tanezumab (2.5–5 mg)/Fasinumab (1–9 mg)	III	↓ WOMAC-pain: 0.84–1.03 points (Tanezumab)/2.7–3.4 points (Fasinumab)	RPOA: 1.4–2.8% (Tanezumab)/5% (Fasinumab); paresthesia ∼ 4.6%; subchondral insufficiency fractures ∼ 1.8%^159^
	capsaicin (CNTX-4975, 1 mg)	II	↓ walking pain (AUC): 1.6 points at 12 weeks; 1.4 points at 24 weeks; ≥50% of patients achieved ≥50% pain reduction (NNT 3.6)	mild-moderate AEs were similar to placebo^160^
bone and cartilage inflammation	Sprifermin (rhFGF18, 100 μg)	II	↑ cartilage thickness +0.05 mm at 2 years, maintained at 5 years; ↓ 10 points WOMAC-pain in predefined high-risk subgroup	no increase in adverse events over 5 years^161^
	Lorecivivint (SM04690, 0.07 mg)	IIb	↓ WOMAC-pain (*p* = 0.04) and ↓ WOMAC-function (*p* = 0.021) at 12 weeks	well tolerated; no apparent joint or systemic toxicity^162^
immunomodulation	FX006 (TA-ER, 32 mg)	II	≥50% ↓ daily pain and WOMAC-A for 16 weeks postinjection	similar to the safety of immediate-release triamcinolone^163^
	Lutikizumab (ABT-981) 25, 100, or 200 mg SC every 2 weeks for up to 50 weeks	II (NCT02087904)	significant WOMAC pain reduction vs placebo at week 16 only with 100 mg (*P* = 0.050); no differences at weeks 26 or 52, nor with 25 mg or 200 mg	injection-site reactions; neutropenia (with some discontinuations); overall manageable^164^
senescence and aging	UBX0101 (4 mg IA)	II	no improvement in WOMAC vs placebo at 12 weeks	well tolerated; no serious treatment-related events^165^
gene	TG-C (TGF-β1-expressing chondrocytes)	III	+15 points IKDC; −25 mm VAS at 12 months (*p* < 0.001)	mild-moderate AEs in 63% vs 44% placebo^166^

a WOMAC: Western Ontario and McMaster Universities Osteoarthritis Index; RPOA: rapidly progressive osteoarthritis; AUC: area under the curve; NNT: number needed to treat; AE: adverse event; IA: intra-articular; IKDC: International Knee Documentation Committee; VAS: visual analog scale; KOOS: Knee Injury and Osteoarthritis Outcome Score.

Tanezumab and Fasinumab, anti-NGF antibodies for OA, demonstrate analgesic efficacy but pose significant risks to joint safety. Tanezumab (IgG2) reduces Western Ontario and McMaster Universities pain by 0.84–1.03 points versus placebo, comparable to NSAIDs, yet causes rapidly progressive osteoarthritis (RPOA) in 1.4–2.8% of patients and paresthesia in 4.6%. These risks are exacerbated by concurrent NSAIDs and prompted FDA rejection in 2021. Fasinumab (IgG4), while more potent (2.7–3.4-point Western Ontario and McMaster Universities reduction), exhibits higher RPOA incidence (5%) and subchondral insufficiency fractures (1.8%). Both agents share dose-dependent joint safety challenges, confirming their clinical utility requires articular damage mitigation strategies.^159^

Capsaicin is a potent TRPV1 agonist that, when injected intra-articularly at 1 mg (CNTX-4975), rapidly defunctionalizes TRPV1-expressing nociceptors, yielding analgesia lasting weeks to months. In a dose-ranging, randomized, double-blind, placebo-controlled Phase II trial in knee OA, the 1 mg dose produced a least-squares mean difference of 1.6 points reduction in the AUC of “pain on walking” at 12 weeks (*p* < 0.0001 vs placebo), with benefit maintained at 24 weeks (LSMD 1.4 points, *p* = 0.0002); over 60% of patients achieved at least 50% pain reduction (NNT = 3.6), and adverse-event rates were similar across groups.^160^

Sprifermin, an anabolic agent that activates fibroblast growth factor receptor 3 (FGFR3) in chondrocytes to promote cartilage regeneration, demonstrated in the Phase II FGF-18 Osteoarthritis Randomized Trial with Administration of Repeated Doses (FORWARD) trial (*n* = 549) that intra-articular injections of 100 μg every six months produced a mean gain of 0.05 mm in total femorotibial cartilage thickness versus placebo at 2 years (*p* = 0.015), which was maintained at 5 years (0.049 mm; 95% CI 0.00–0.10; *p* = 0.015).^161^ Although there was no overall symptomatic improvement, a predefined risk subgroup (baseline joint space width 1.5–3.5 mm; Western Ontario and McMaster Universities pain 40–90) experienced an additional 10.08-point reduction in Western Ontario and McMaster Universities pain (95% CI 5.53–25.68) and had zero arthroplasties by year 5 versus 4.6% in the placebo group.^167^

A new Wnt pathway modulator, lorecivivint (SM04690), demonstrated clinically meaningful improvements in pain and function in a 24 week Phase 2b trial, with the 0.07 mg dose significantly reducing Western Ontario and McMaster Universities Pain and Function scores versus placebo at week 12 (*p* = 0.04 and *p* = 0.021, respectively) and selecting 0.07 mg for ongoing Phase III studies.^162^ FX006 (TA-ER), an extended-release triamcinolone acetonide microsphere formulation, has consistently achieved ≥50% reductions from baseline in average daily pain and Western Ontario and McMaster Universities-A through 16 weeks postinjection versus saline placebo in randomized trials, with a safety profile comparable to immediate-release suspension.^163^

Lutikizumab (ABT-981) is a dual-variable-domain immunoglobulin that neutralizes both interleukin-1 alpha (IL-1α) and IL-1β. A 2020 Bayesian network meta-analysis of knee OA trials found no significant benefit over placebo for either pain relief (SMD 1.11; 95% CI −2.29 to 4.52) or functional improvement (SMD 0.99; 95% CI −0.43 to 4.25). In the phase II trial conducted by Unity Biotechnology in patients with knee OA, a single 4 mg intra-articular dose of the senolytic UBX0101, was well tolerated but failed to improve Western Ontario and McMaster Universities function scores at 12 weeks versus placebo (*n* = 183).^165^

In a multicenter, randomized, double-blind, placebo-controlled Phase III trial (*n* = 159), a single injection of TissueGene-C (TG-C) produced a 15-point improvement in the International Knee Documentation Committee (IKDC) score and a 25 mm reduction in the visual analog scale at 12 months versus 5 points and 10 mm with placebo (*p* < 0.001); these effects were evident and sustained at months 6 and 9. Both Western Ontario and McMaster Universities, and the Knee Injury and Osteoarthritis Outcome Score (KOOS), favored TG-C (*p* ≤ 0.003), with trends toward reduced bone area and lower C-terminal cross-linked telopeptide of type I collagen (CTX-I). Treatment-related adverse events, mostly mild, occurred in 63% of TG-C patients versus 44% of placebo patients.^166^

## Conclusions

This comprehensive review critically examines the evidence for HA, CS, and GlcN as widely used therapeutic options in OA management, while acknowledging the significant controversies surrounding their efficacy. The evidence indicates that their benefits are most pronounced when utilizing pharmaceutical-grade formulations (pCS, pCGS, and high-molecular-weight HA), which mitigate the widespread variability inherent in nutraceutical products. Among these, pharmaceutical-grade CS emerges with the most robust evidence for long-term, disease-modifying effects, while intra-articular HA and oral CS/GlcN combinations remain the most prevalent clinical choices due to their synergistic potential and favorable risk-benefit profile. However, the translation of this potential into consistent clinical outcomes is hampered by two fundamental challenges: the critical issue of product heterogeneity, which leads to erratic bioavailability and efficacy, and a disruptive regulatory discrepancy that classifies intra-articular HA as a medical device and CS/GlcN as dietary supplements, thereby bypassing the stringent efficacy and quality controls required for pharmaceutical drugs.

These challenges directly contribute to the contradictory findings in literature and clinical practice, stifling broader acceptance. To propel the field forward and translate mechanistic insights into tangible clinical benefits, future efforts must be directed toward personalized medicine through biomarker discovery, advanced delivery system engineering, rational combination therapies, and definitive long-term structural studies using quantitative imaging. Addressing the current limitations requires a concerted multistakeholder approach: regulatory agencies must re-evaluate classification guidelines, clinicians must be educated on critical differences between prescription and over-the-counter compounds, and researchers must adopt standardized measures and study designs that explain interindividual variability. In conclusion, while SYSADOAs represent a valuable and safe arsenal in the fight against OA, their full potential remains largely untapped. The path forward requires a paradigm shift from generic application to a precision medicine approach, fueled by standardized manufacturing, innovative delivery solutions, and robust regulatory frameworks to finally harness the true promise of these agents to modify disease progression and improve the quality of life for millions of OA patients worldwide.

## Abbreviations

CIA: collagen-induced arthritis
COX-2: cyclooxygenase 2
CS: chondroitin sulfate
ECM: extracellular matrix
ERK1/2: extracellular signal-regulated kinases 1 and 2
ESCEO: European Society for Clinical and Economic Aspects of Osteoporosis, Osteoarthritis and Musculoskeletal Diseases
EULAR: European League against Rheumatism
GAG: glycosaminoglycan
GalNAc: *N*-acetylgalactosamine
GH: glucosamine hydrochloride
GlcN: glucosamine
GS: glucosamine sulfate
FDA: Food and Drug Administration
HA: hyaluronic acid
hOCS: human primary osteoclasts
HWM-HA: high molecular weight hyaluronic acid
ICAM-1: intercellular adhesion molecule 1
IFN-&gamma: interferon-gamma
IL-1&beta: interleukin-1β
IL-1&beta: interleukin 1 beta
IL-6: interleukin-6
INOs: inducible nitric oxide synthase
JNK: c-Jun *N*-terminal kinases
LAYN: Layilin
LMW-HA: low molecular weight hyaluronic acid
LPS: lipopolysaccharide
MIA: monosodium iodoacetate
MMP-1: matrix metalloproteinase-1
MMP-13: matrix metalloproteinase-13
MMPs: matrix metalloproteinases
MMWHA: medium molecular weight hyaluronic acid
NF-κB: factor nuclear kappa B
NO: nitric oxide
NOS-2: nitric oxide synthase 2
NSAIDs: nonsteroidal anti-inflammatory drugs
OA: osteoarthritis
p38MAPK: p38 mitogen-activated protein kinase
PAMPs: pathogen-associated molecular patterns
pCGS: prescription-grade crystalline glucosamine sulfate
pCS: pharmaceutical grade formulation of chondroitin sulfate
PGE2: prostaglandin E_2_
PS: sulfated polysaccharide
RA: rheumatoid arthritis
RCT: randomized clinical trial
RHAMM: receptor for hyaluronic acid-mediated motility
ROS: reactive oxygen species
SOD: superoxide dismutase
SYSADOAs: symptomatic slow-acting drugs for osteoarthritis
TLR-2/4: toll-like receptor 2 and 4
TNF-&alpha: tumor necrosis factor alpha
TNF-α: tumor necrosis factor alpha
UHMW-HA: ultrahigh molecular weight hyaluronic acid
